# Singular Case of Twins

**Published:** 1847-04

**Authors:** 


### ARTICLE XI.

### SINGULAR CASE OF TWINS.

Dr. J. Heath, of Janesville, W. T., in a letter, gives the following interesting case.

While in attendance upon a lady in labor with her first child, about a year ago, he observed a tumor at the umbilicus that attracted his attention. It had not been previously observed. He made pressure upon it, and as the child advanced it subsided. The child was born, and the placenta expelled from the uterus and removed from the vagina. On examining the after-birth it was found that the membranes were thickened, and formed a sack in which was found a fœtus about eight inches long and natural, except the head was flattened between the parietal bones, so as almost to destroy the transverse diameter. There was no appearance of decomposition.

A case of twins in which monstrosity prevented the development of one child, which formed the tumor above referred to. The mother and child did well.

